# Supplementary material for: Infrared thermal imaging monitoring on hands when performing repetitive tasks: An experimental study
Source: PLoS One. 2021 May 12;16(5):e0250733. doi: 10.1371/journal.pone.0250733 (PMC8115808; doi:10.1371/journal.pone.0250733)
Supplement: S1 Annexure — (DOCX) [file pone.0250733.s001.docx]

**S1 Annexure.** Nomenclature of variables

| DF1R_0 | Dorsal Finger Thumb Right_0 Minutes | PF1R_0 | Palm Finger Thumb Right_0 Minutes |
| --- | --- | --- | --- |
| DF2R_0 | Dorsal Finger Index Right_0 Minutes | PF2R_0 | Palm Finger Index Right_0 Minutes |
| DF3R_0 | Dorsal Finger Middle Right_0 Minutes | PF3R_0 | Palm Finger Middle Right_0 Minutes |
| DF4R_0 | Dorsal Finger Ring Right_0 Minutes | PF4R_0 | Palm Finger Ring Right_0 Minutes |
| DF5R_0 | Dorsal Finger Little Right_0 Minutes | PF5R_0 | Palm Finger Little Right_0 Minutes |
| DF1L_0 | Dorsal Finger Thumb Left_0 Minutes | PF1L_0 | Palm Finger Thumb Left_0 Minutes |
| DF2L_0 | Dorsal Finger Index Left_0 Minutes | PF2L_0 | Palm Finger Index Left_0 Minutes |
| DF3L_0 | Dorsal Finger Middle Left_0 Minutes | PF3L_0 | Palm Finger Middle Left_0 Minutes |
| DF4L_0 | Dorsal Finger Ring Left_0 Minutes | PF4L_0 | Palm Finger Ring Left_0 Minutes |
| DF5L_0 | Dorsal Finger Little Left_0 Minutes | PF5L_0 | Palm Finger Little Left_0 Minutes |
| DF1R_10 | Dorsal Finger Thumb Right_10 Minutes | PF1R_10 | Palm Finger Thumb Right_10 Minutes |
| DF2R_10 | Dorsal Finger Index Right_10 Minutes | PF2R_10 | Palm Finger Index Right_10 Minutes |
| DF3R_10 | Dorsal Finger Middle Right_10 Minutes | PF3R_10 | Palm Finger Middle Right_10 Minutes |
| DF4R_10 | Dorsal Finger Ring Right_10 Minutes | PF4R_10 | Palm Finger Ring Right_10 Minutes |
| DF5R_10 | Dorsal Finger Little Right_10 Minutes | PF5R_10 | Palm Finger Little Right_10 Minutes |
| DF1L_10 | Dorsal Finger Thumb Left_10 Minutes | PF1L_10 | Palm Finger Thumb Left_10 Minutes |
| DF2L_10 | Dorsal Finger Index Left_10 Minutes | PF2L_10 | Palm Finger Index Left_10 Minutes |
| DF3L_10 | Dorsal Finger Middle Left_10 Minutes | PF3L_10 | Palm Finger Middle Left_10 Minutes |
| DF4L_10 | Dorsal Finger Ring Left_10 Minutes | PF4L_10 | Palm Finger Ring Left_10 Minutes |
| DF5L_10 | Dorsal Finger Little Left_10 Minutes | PF5L_10 | Palm Finger Little Left_10 Minutes |
| DF1R_15 | Dorsal Finger Thumb Right_15 Minutes | PF1R_15 | Palm Finger Thumb Right_15 Minutes |
| DF2R_15 | Dorsal Finger Index Right_15 Minutes | PF2R_15 | Palm Finger Index Right_15 Minutes |
| DF3R_15 | Dorsal Finger Middle Right_15 Minutes | PF3R_15 | Palm Finger Middle Right_15 Minutes |
| DF4R_15 | Dorsal Finger Ring Right_15 Minutes | PF4R_15 | Palm Finger Ring Right_15 Minutes |
| DF5R_15 | Dorsal Finger Little Right_15 Minutes | PF5R_15 | Palm Finger Little Right_15 Minutes |
| DF1L_15 | Dorsal Finger Thumb Left_15 Minutes | PF1L_15 | Palm Finger Thumb Left_15 Minutes |
| DF2L_15 | Dorsal Finger Index Left_15 Minutes | PF2L_15 | Palm Finger Index Left_15 Minutes |
| DF3L_15 | Dorsal Finger Middle Left_10 Minutes | PF3L_15 | Palm Finger Middle Left_10 Minutes |
| DF4L_15 | Dorsal Finger Ring Left_15 Minutes | PF4L_15 | Palm Finger Ring Left_15 Minutes |
| DF5L_15 | Dorsal Finger Little Left_15 Minutes | PF5L_15 | Palm Finger Little Left_15 Minutes |
| DF1R_20 | Dorsal Finger Thumb Right_20 Minutes | PF1R_20 | Palm Finger Thumb Right_20 Minutes |
| DF2R_20 | Dorsal Finger Index Right_20 Minutes | PF2R_20 | Palm Finger Index Right_20 Minutes |
| DF3R_20 | Dorsal Finger Middle Right_20 Minutes | PF3R_20 | Palm Finger Middle Right_20 Minutes |
| DF4R_20 | Dorsal Finger Ring Right_20 Minutes | PF4R_20 | Palm Finger Ring Right_20 Minutes |
| DF5R_20 | Dorsal Finger Little Right_20 Minutes | PF5R_20 | Palm Finger Little Right_20 Minutes |
| DF1L_20 | Dorsal Finger Thumb Left_20 Minutes | PF1L_20 | Palm Finger Thumb Left_20 Minutes |
| DF2L_20 | Dorsal Finger Index Left_20 Minutes | PF2L_20 | Palm Finger Index Left_20 Minutes |
| DF3L_20 | Dorsal Finger Middle Left_20 Minutes | PF3L_20 | Palm Finger Middle Left_20 Minutes |
| DF4L_20 | Dorsal Finger Ring Left_20 Minutes | PF4L_20 | Palm Finger Ring Left_20 Minutes |
| DF5L_20 | Dorsal Finger Little Left_20 Minutes | PF5L_20 | Palm Finger Little Left_20 Minutes |
