# Supplementary material for: Infrared thermal imaging monitoring on hands when performing repetitive tasks: An experimental study
Source: PLoS One. 2021 May 12;16(5):e0250733. doi: 10.1371/journal.pone.0250733 (PMC8115808; doi:10.1371/journal.pone.0250733)
Supplement: S2 Annexure — (DOCX) [file pone.0250733.s002.docx]

**S2 Annexure.** Results of the normality test Anderson Darling

| DORSAL | | PALMAR | |
| --- | --- | --- | --- |
| Variable | P-value | Variable | P-value |
| DF1R_0 | 0.035 | PF1R_0 | 0.181 |
| DF2R_0 | 0.039 | PF2R_0 | 0.084 |
| DF3R_0 | 0.065 | PF3R_0 | 0.021 |
| DF4R_0 | 0.283 | PF4R_0 | 0.106 |
| DF5R_0 | 0.542 | PF5R_0 | 0.386 |
| DF1L_0 | 0.045 | PF1L_0 | 0.283 |
| DF2L_0 | 0.090 | PF2L_0 | 0.092 |
| DF3L_0 | 0.140 | PF3L_0 | 0.027 |
| DF4L_0 | 0.219 | PF4L_0 | 0.057 |
| DF5L_0 | 0.542 | PF5L_0 | 0.579 |
| **DF1R_10** | 0.005 | **PF1R_10** | 0.005 |
| DF2R_10 | 0.009 | **PF2R_10** | 0.005 |
| DF3R_10 | 0.035 | **PF3R_10** | 0.005 |
| DF4R_10 | 0.059 | PF4R_10 | 0.006 |
| DF5R_10 | 0.109 | PF5R_10 | 0.049 |
| **DF1L_10** | 0.005 | **PF1L_10** | 0.005 |
| **DF2L_10** | 0.005 | **PF2L_10** | 0.005 |
| DF3L_10 | 0.135 | PF3L_10 | 0.031 |
| DF4L_10 | 0.118 | PF4L_10 | 0.122 |
| DF5L_10 | 0.259 | PF5L_10 | 0.107 |
| **DF1R_15** | 0.005 | **PF1R_15** | 0.005 |
| **DF2R_15** | 0.005 | **PF2R_15** | 0.005 |
| DF3R_15 | 0.048 | PF3R_15 | 0.015 |
| DF4R_15 | 0.030 | PF4R_15 | 0.026 |
| DF5R_15 | 0.156 | PF5R_15 | 0.07 |
| DF1L_15 | 0.005 | **PF1L_15** | 0.005 |
| DF2L_15 | 0.005 | **PF2L_15** | 0.005 |
| DF3L_15 | 0.059 | PF3L_15 | 0.027 |
| DF4L_15 | 0.118 | PF4L_15 | 0.072 |
| DF5L_15 | 0.204 | PF5L_15 | 0.24 |
| DF1R_20 | 0.005 | **PF1R_20** | 0.005 |
| DF2R_20 | 0.005 | **PF2R_20** | 0.005 |
| DF3R_20 | 0.033 | **PF3R_20** | 0.005 |
| DF4R_20 | 0.103 | **PF4R_20** | 0.005 |
| DF5R_20 | 0.185 | PF5R_20 | 0.073 |
| DF1L_20 | 0.005 | **PF1L_20** | 0.005 |
| DF2L_20 | 0.005 | **PF2L_20** | 0.005 |
| DF3L_20 | 0.066 | **PF3L_20** | 0.005 |
| DF4L_20 | 0.057 | PF4L_20 | 0.007 |
| DF5L_20 | 0.142 | PF5L_20 | 0.097 |
