# Supplementary material for: Infrared thermal imaging monitoring on hands when performing repetitive tasks: An experimental study
Source: PLoS One. 2021 May 12;16(5):e0250733. doi: 10.1371/journal.pone.0250733 (PMC8115808; doi:10.1371/journal.pone.0250733)
Supplement: S3 Annexure — (DOCX) [file pone.0250733.s003.docx]

**S3 Annexure.** Mann-Whitney U test between ROIs and the sex variable

| Variable | Sig. | Variable | Sig. | Variable | Sig. | Variable | Sig. |
| --- | --- | --- | --- | --- | --- | --- | --- |
| DF1R_0 | 0.001* | DF1R_15 | 0* | PF1R_0 | 0.001* | PF1R_15 | 0.002* |
| DF2R_0 | 0.001* | DF2R_15 | 0.002* | PF2R_0 | 0.001* | PF2R_15 | 0.003* |
| DF3R_0 | 0.003* | DF3R_15 | 0* | PF3R_0 | 0.001* | PF3R_15 | 0.001* |
| DF4R_0 | 0.003* | DF4R_15 | 0* | PF4R_0 | 0.002* | PF4R_15 | 0.001* |
| DF5R_0 | 0.004* | DF5R_15 | 0.001* | PF5R_0 | 0.002* | PF5R_15 | 0.003* |
| DF1L_0 | 0* | DF1L_15 | 0* | PF1L_0 | 0* | PF1L_15 | 0* |
| DF2L_0 | 0.001* | DF2L_15 | 0.001* | PF2L_0 | 0* | PF2L_15 | 0* |
| DF3L_0 | 0* | DF3L_15 | 0.001* | PF3L_0 | 0* | PF3L_15 | 0* |
| DF4L_0 | 0* | DF4L_15 | 0.001* | PF4L_0 | 0.001* | PF4L_15 | 0.001* |
| DF5L_0 | 0.001* | DF5L_15 | 0.002* | PF5L_0 | 0* | PF5L_15 | 0.001* |
| DF1R_10 | 0* | DF1R_20 | 0.005* | PF1R_10 | 0* | PF1R_20 | 0.011* |
| DF2R_10 | 0.003* | DF2R_20 | 0.001* | PF2R_10 | 0.001* | PF2R_20 | 0.004* |
| DF3R_10 | 0.003* | DF3R_20 | 0.003* | PF3R_10 | 0.001* | PF3R_20 | 0.001* |
| DF4R_10 | 0.005* | DF4R_20 | 0.005* | PF4R_10 | 0.001* | PF4R_20 | 0.005* |
| DF5R_10 | 0.005* | DF5R_20 | 0.008* | PF5R_10 | 0.002* | PF5R_20 | 0.022* |
| DF1L_10 | 0* | DF1L_20 | 0* | PF1L_10 | 0* | PF1L_20 | 0.001* |
| DF2L_10 | 0.001* | DF2L_20 | 0.001* | PF2L_10 | 0.002* | PF2L_20 | 0.002* |
| DF3L_10 | 0.001* | DF3L_20 | 0.001* | PF3L_10 | 0.001* | PF3L_20 | 0.001* |
| DF4L_10 | 0* | DF4L_20 | 0.001* | PF4L_10 | 0* | PF4L_20 | 0.001* |
| DF5L_10 | 0.001* | DF5L_20 | 0.006* | PF5L_10 | 0.002* | PF5L_20 | 0.006* |
