# Supplementary material for: Infrared thermal imaging monitoring on hands when performing repetitive tasks: An experimental study
Source: PLoS One. 2021 May 12;16(5):e0250733. doi: 10.1371/journal.pone.0250733 (PMC8115808; doi:10.1371/journal.pone.0250733)
Supplement: Attachment — Submitted filename: Responce to reviwer.docx [file pone.0250733.s004.docx]

**Reply to the reviewer**

Dear reviewer, we appreciate your comments for improve this paper. In following paragraphs, your comments are in bold and our response in italic.

**The paper was improved, and authors replied to most o of the issues raised by the reviewers. Beside this consideration I suggest additional changes before the publication of the paper.**
*Thanks a lot for that comment. We have attended yours comments and hope to fulfill your expectations.*

**Authors added several huge tables without a specific reason, please remove them or move to appendix or additional materials. Two-page table is almost illegible.**

*Considering your suggestion, tables were eliminated and in current version of the paper, three tables are reported as annex or supporting material.*

**-For instance, I suggest removing table 3, or move to the additional materials, since for the comprehension of the results it is enough to explain for which variables normality was tested and then report that for normal variables it was used a certain parametric test, for those not normal it was used a non-parametric test adding which one and why.**

*R= Your suggestion was considered and table 3 was removed and appears as supporting material.*

**What happened to normal variables? I couldn’t find any consideration about them.**

*R= They were analyzed through non-parametric tests, no special treatment was given to these variables since all the analysis was carried out together with ROIs and variables.*

**-I would move also tab 2 in appendix or** **additional materials.**

*R= Table 2 was moved to supporting material*

**-I found that authors mixed methods and results, for instance I suggest to move tab 5 with Mann Whitney in results section and I suggest also to replace the content of the table with a self-explanatory graph. But most of the data put in section 2 should be moved to section 3.**

*R=Statistical analyzes were changed to section 3 of results and referred to Annex 3 because is too long and difficult to fit in only one page.*

*Also in this current version, Figure 4 was added because in more self-explanatory. Figure 3 appears as follow.*

**-In table 7 I also suggest to report the content of the table with a graph, for instance using x for time and y for ROIs T, one for the dorsal and the other for the palm.**

*R=This observation was addressed and Figure 4 was added as follow.*

**-It would be useful to see an image with the ROIs positioned on the hands to see where the temperature extraction was made, adding also the average number of pixel considered in the ROIs**

*R= In figure two, each of the ROIs can be seen delimited. The pixel of the camera are in L174,* *the average number pixel in ROIs is 2x3*

**-Table 8: replace temperature gradient with and add if the data are statistically significant adding a “*” for those or a column with p-values.**

*R=In table 8 temperature gradient was replaced with temperature differences and table 9, and adding * for values whit with p<0.05*

**-Line 271: replace text with test**

*R= The word text was replaced for test*

**-Line 272 replace “To test the relationship between all variables” with “To test the effect of…”**

*R=The text was replaced whit “To test the effect of…”*

**-Line 273: what the authors mean with “sport” it is a very generic term.**

*R= Thanks for that observation. The text sport was replaced whit participants that practice some sports*

**-Line 279 add to ROIs “temperature”**

*R= Thanks for that comment. That word was add*

**-How BMI was used to divide the two groups?**

*R= The database used for the statistical analysis divided by the sex of the participants*
