# Supplementary material for: Infrared thermal imaging monitoring on hands when performing repetitive tasks: An experimental study
Source: PLoS One. 2021 May 12;16(5):e0250733. doi: 10.1371/journal.pone.0250733 (PMC8115808; doi:10.1371/journal.pone.0250733)
Supplement: Attachment — Submitted filename: Response to Reviewers.docx [file pone.0250733.s005.docx]

Response to Editor

Dear editor, we appreciate your comments and recommendations for improve our paper. In current version, the track change in word has been activated and you can review easier.

The following paragraphs contain your comments in bold type, while our responses are in italics. Here is important to mention that all Figures has been tested and passed the Preflight Analysis and Conversion Engine (PACE) digital diagnostic.

**This manuscript is nearly ready for publication and was not sent out to reviewers. In a journal with a post-acceptance proofing process, this might be an acceptance. However, there are some issues that should be addressed before this manuscript is publication ready.**

1. **Please provide (in the text) a full clarification and explanation of the reviewer's last comment: how BMI was used.**

***Our response.*** *Thanks a lot for that comment. After a review, we see that translator keep the acronym IMC in Spanish and in current version of the paper we change everything to BMI (Body Mass Index). Lines 276 – 277 indicates that variables as age, BMI, fractures, and sports do not have an influence on ROI temperature and that is why we do not report tables referring BMI.*

**2) Please provide more detailed figure legends that allow the reader to understand the figures without needing to dig into the text. (I phrase this in terms of "figure legends" but may require alterations to the figures as well.) In general, figure legends should describe the figure's key finding/conclusion (ideally as the opening sentence of the legend). In addition, specifically:**

**Figure 1: The legend should contain the text in lines 246-247.**

***Our response.*** *The legend for Figure 1 was replaced by: Steps of activity that participants have carried out. The process consisted of winding cables without stopping for 10 minutes.*

**Figure 2: Legend should contain the text in lines 249-251. Should be written to clarify that the "2x3" number applies to the tear ducts, not the fingers.**

Our response. The legend for Figure 2 was replaced by: *Thermogram of face and palm of the hand with their respective region of interest. The ROIs for face were the carotids, which are found in the tear duct of the eye and ROIs for hands were each region delimited by fingers. Resolution average was 160 x 120 pixels.*

**Figure 3: The text on lines 281-285 does not seem to match the figure or figure legend. For this figure, it ought to indicate which points meet the significance threshold, and its legend ought to explain key abbreviations. However, my suspicion is that this figure is one that has been pushed to Supplementary Material, and that you should remove references "Figure 3" in 281-285. The real "Temperature of the fingers of the dorsal hand" figure is missing.**

***Our response:*** *Thanks for that comment. In current version of the paper, it is indicated that all variables were statistically significant in line 288. The abbreviations were explained in lines 284-286.The name for Figure 3 was changed to: Two-tailed hypothesis test at 95% confidence level for ROIs variables, and now is according to the text in paragraph.*

**Figure 4: The horizontal axis should be clarified - it is unclear to me how those numbers work.**

***Our response:*** *Thanks a lot for that comment. Current version of Figure 4 has been adapted and x-axis was modified as follow.*

**Figure 5: 5a/5b either need to be combined into a single image containing both charts or be separated out fully into two figures (with separate numbers and legends).**

***Our response:*** *Thanks a lot for that comment. Figure 5a now is Figure 5 and Figure 5b now is Figure 6, and both have different legend. The call-in text also was modified.*

**Figure 6: 6a/6b as above.**

***Our response:*** *The graphs were separated. Figure 6a in current version is Figure 7 and Figure 6b is now Figure 8. The call-in text also was modified.*
